# Diabetic Retinopathy Grading by Deep Graph Correlation Network on Retinal Images Without Manual Annotations

**DOI:** 10.3389/fmed.2022.872214

**Published:** 2022-04-14

**Authors:** Guanghua Zhang, Bin Sun, Zhixian Chen, Yuxi Gao, Zhaoxia Zhang, Keran Li, Weihua Yang

**Affiliations:** ^1^Department of Intelligence and Automation, Taiyuan University, Taiyuan, China; ^2^Graphics and Imaging Laboratory, University of Girona, Girona, Spain; ^3^Shanxi Eye Hospital, Taiyuan, China; ^4^Shanxi Finance and Taxation College, Taiyuan, China; ^5^The Laboratory of Artificial Intelligence and Bigdata in Ophthalmology, The Affiliated Eye Hospital of Nanjing Medical University, Nanjing, China

**Keywords:** diabetic retinopathy, retinal image classification, graph correlation network, unsupervised learning, automated diagnosis

## Abstract

**Background:**

Diabetic retinopathy, as a severe public health problem associated with vision loss, should be diagnosed early using an accurate screening tool. While many previous deep learning models have been proposed for this disease, they need sufficient professional annotation data to train the model, requiring more expensive and time-consuming screening skills.

**Method:**

This study aims to economize manual power and proposes a deep graph correlation network (DGCN) to develop automated diabetic retinopathy grading without any professional annotations. DGCN involves the novel deep learning algorithm of a graph convolutional network to exploit inherent correlations from independent retinal image features learned by a convolutional neural network. Three designed loss functions of graph-center, pseudo-contrastive, and transformation-invariant constrain the optimisation and application of the DGCN model in an automated diabetic retinopathy grading task.

**Results:**

To evaluate the DGCN model, this study employed EyePACS-1 and Messidor-2 sets to perform grading results. It achieved an accuracy of 89.9% (91.8%), sensitivity of 88.2% (90.2%), and specificity of 91.3% (93.0%) on EyePACS-1 (Messidor-2) data set with a confidence index of 95% and commendable effectiveness on receiver operating characteristic (ROC) curve and t-SNE plots.

**Conclusion:**

The grading capability of this study is close to that of retina specialists, but superior to that of trained graders, which demonstrates that the proposed DGCN provides an innovative route for automated diabetic retinopathy grading and other computer-aided diagnostic systems.

## Introduction

Diabetic retinopathy, which has become a severe public health problem, is estimated to affect 200 million patients in the next two decades (1–4). Diabetic retinopathy is a severe etiology of vision loss, possibly causing blindness, which can be avoided by early diagnosis, fundus screening, and temporal treatment (4). Fundus images with professional interpretation are a commonly accepted screening strategy for blindness prevention, with superior capability for in-person dilated eye examinations (5). However, the procedures for observing diabetic retinopathy confront challenges by factors correlated to execution, availability of professionals, and substantial financial sustainability (6–9). The automatic grading of diabetic retinopathy appears to have potential advantages in solving these obstacles, such as increasing efficiency, scalability, and coverage of examining procedures, extending applications in developed regions, and improving patient prevention by providing early diagnosis and referral.

To take full advantage of the clinical application of automatic grading, deep learning technology has been employed to develop a grading system for diabetic retinopathy and achieve considerable progress (10–12). Deep learning adopts artificial intelligence and representation learning tools to address large amounts of data and informative features (13, 14). Recent studies (15–17) have elaborated highly accurate sensitivity and specificity (>90%) in the automatic detection of diabetic retinopathy from fundus images, principally involving accurately annotated fundus images from experienced and professional experts. Recently, the performance of deep learning systems in the examination of diabetic retinopathy is close to that of expert-level diagnoses for grading fundus images, with the help of extensive labeled fundus images from accurate grading by professional manpower. In clinical grading, concentrating extensive professional human resources to provide annotations for developing deep learning systems is unreasonable. Therefore, the performance of an ideal automatic system should also have an excellent capability for detecting diabetic retinopathy from fundus images without manual annotations.

This study mainly aimed to explore the automatic diabetic retinopathy grading without requiring manual annotations on fundus images. This is developed from advanced deep learning technology, thereby saving large amounts of expensive and time-consuming professional manpower. Furthermore, extensive validations on two publicly available diabetic retinopathy grading datasets [EyePACS-1 (18) and Messidor-2 (19)], which consist of various severe clinical challenges, demonstrate the accessibility for automatic grading without manual annotations.

The automatic diabetic retinopathy grading system aims to assist in early diagnosis and grading, which is crucial in diabetic retinopathy screening procedures and requires robust discernment and sufficiently wide computer screens. Several representative attempts have achieved promising effectiveness for grading fundus images (1, 7, 8, 10, 19).

Specifically, Abràmoff et al. (20) developed a deep learning enhanced algorithm for the automatic grading of diabetic retinopathy, and it achieved significantly better performance [sensitivity, 96.8%; specificity, 87.0%; and area under the curve (AUC), 0.980% on MESSIDOR-2 dataset] than did previous methods. Gulshan et al. (18) applied a specific type of neural network optimized for image classification using a retrospective development dataset of 128,175 retinal images for automated grading of diabetic retinopathy and diabetic macular oedema in a retinal fundus photograph. This maintained a sensitivity, specificity, and an AUC of 90.3, 98.1, and 0.991% on EyePACS-1 dataset and 87.0, 98.5, and 0.990% on MESSIDOR-2 dataset, respectively. Ting et al. (14) evaluated the performance of a deep learning system for diabetic retinopathy and related eye diseases using 494,661 retinal images, and it obtained a sensitivity of 95.0%, specificity of 89.7%, and an AUC of 0.941 for overall diabetic retinopathy grading. Gulshan et al. (21) generalized the deep learning automated diabetic retinopathy system to a population of Indian patients at Aravind and Sankara Nethralaya, achieving a sensitivity of 88.9 and 92.1%, specificity of 92.2 and 95.2%, and an AUC of 0.963 and 0.980 for the Aravind Eye Hospital and Sankara Eye Hospital datasets, respectively. This demonstrates the feasibility of using an automated grading system to expand screening programs. Kathiresan et al. (22) proposed a synergic deep learning model for the automated grading and classification of fundus diabetic retinopathy images, involving various processes of pre-processing, segmentation, and classification. This exhibited an excellent accuracy of 99.28%, sensitivity of 98.54%, and specificity of 99.38% on the Messidor dataset.

Although previous deep learning grading systems demonstrated excellent performance on publicly available or self-collected fundus image data, they require expensive and time-consuming professional labor to annotate large amounts of retinal images to guide deep learning models. That severely restricts the application ability of the deep learning models in clinical practice, leading to the decrease of detection efficiency, and patients can not get timely diagnosis and delay the condition. As such, an excellent diabetic retinopathy grading system should be able to handle fundus image grading without the need for manual annotations to provide flexibility in clinical applications.

## Materials and Methods

To support this development, the EyePACS dataset, which was retrospectively collected from the Eye Picture Archive and Communication System (EyePACS) in the United States and three eye hospitals in India (Aravind Eye Hospital, Sankara Nethralaya, and Narayana Nethralaya), provides a large-scale set of high-resolution retinal images obtained under a variety of imaging conditions. EyePACS is a flexible protocol and web-based telemedicine system for diabetic retinopathy screening and collaboration among clinicians. All images from EyePACS were obtained from different models and types of cameras, and were de-identified according to the Health Insurance Portability and Accountability Act Safe Harbor prior to transferring to the study investigators. Ethics review and institutional review board exemptions were obtained using the Quorum Review IRB. To implement the clinical validation, we randomly sampled 10,286 retinal macula-centered images from 5,158 patients using the EyePACS data (23), wherein different models and types of cameras affected the visual appearance of the left vs. right eyes of the patients. Some images are shown as one would see the retina anatomically (macula on the left and optic nerve on the right for the right eye). Others are shown as one would see through a microscope condensing lens (i.e., inverted, as one sees in a typical live eye exam).

Furthermore, this prospective study enrolled a second dataset, Messidor-2 (19), to prove the scalability of DGCN. Messidor-2 collected diabetic retinopathy examinations with two macula-centered eye fundus images from each eye, part of which was provided by the Messidor program partners and others were from the Messidor extension of previously published examinations from Brest University Hospital. In contrast to the original Messidor dataset of 1,200 images, it consists of 1,748 digital retinal color images from 874 patients with diabetes. Patients with diabetes were recruited from the Ophthalmology Department of Brest University Hospital (France) between October 16, 2009 and September 6, 2010. Eye fundi were imaged, without pharmacological dilation, using a Topcon TRC NW6 non-mydriatic fundus camera with a 45-degree field of view. Only macula-centered images were included in the dataset. Messidor-Extension contains 345 examinations (690 images in JPG format). Other descriptions of this dataset can be found in (19).

All images in the clinical validation sets were graded by several ophthalmologists for the presence of diabetic retinopathy. Diabetic retinopathy severity is annotated as none, mild, moderate, severe, or proliferative, according to the International Clinical Retinopathy Scale (24). The severity of each grade is as follows:

• None: no apparent retinopathy (no abnormalities).
• Mild: mild non-proliferative diabetic retinopathy (microaneurysms only).
• Moderate: moderate non-proliferative diabetic retinopathy (more than microaneurysms but less severe non-proliferative diabetic retinopathy).
• Severe: severe non-proliferative diabetic retinopathy (any extensive intraretinal hemorrhages in each of the four quadrants, definite venous beading in 2+ quadrants, prominent IRMA in 1+ quadrant, and no signs of proliferative retinopathy).
• Proliferative: proliferative diabetic retinopathy (one or more neovascularization and vitreous/preretinal hemorrhage).

As introduced in the datasets, the graders were CN-licensed ophthalmologists and had diabetic retinopathy diagnosis experience of at least 5 years. Furthermore, every image was randomly sent to graders with more than three grades for implementing impartial judgment.

### Statistical Analysis

This study first reports the sensitivity and specificity results in 95% confidence intervals (CIs), which are then compared to the diagnosis from retina specialists and trained graders. The sensitivity and specificity are defined as follows:


$$\text{Sensitivity}=\frac{T\,P}{T\,P+F\,N}$$

$$\text{Specificity}=\frac{T\,N}{T\,N+F\,P}$$

where *TP* denotes the number of truly predicted positive samples, *FN* is the number of falsely predicted negative samples, *TN* is the number of truly predicted negative samples, and *FP* is the number of falsely predicted positive samples. To characterize the sensitivity and specificity of DGCN, this study reports the presence of referable diabetic retinopathy (moderate or worse diabetic retinopathy) on EyePACS and Messidor-2 data sets.

Second, this study elaborates on the receiver operating characteristic (ROC) curve and its AUC to measure the performance of DGCN on diabetic retinopathy grading without manual annotations. The ROC curve is a graphical plot that illustrates the diagnostic ability of a binary classifier system, as its discrimination threshold is varied. The AUC measures the entire two-dimensional area underneath the entire ROC curve. ROC is a probability curve, and AUC represents the degree or measure of separability. A higher AUC indicates that the model is better in distinguishing between patients with and without the disease.

Third, an important speculation for DGCN is the t-distributed stochastic neighbour embedding (t-SNE) (25), a technique for dimension reduction that is particularly appropriate for visualizing high-dimensional features. This metric can synthetically perform feature learning ability for deep learning models, and it is widely used in unsupervised frameworks. In addition, we utilized PyTorch and Python to develop our DGCN model and conduct the experiments.

### Development of Deep Learning Algorithm

Deep learning (DL) technology is effective for pattern recognition based on the rapid development of computing power (GPUs), especially for automated diagnosis, such as in previous studies (26–28). In this study, DL involves millions of trainable parameters to achieve the target task using objective functions. It aims to generate the diabetic retinopathy severity for retinal image data from patients, which do not require any manual annotations, rather than requiring large amounts of labeled data as previous time-consuming and cost-expensive DL systems have done (11, 12, 21). These works were developed from an early DL architecture of a convolutional neural network (CNN), which allows learning independent representations from a single image. In addition, an advanced DL framework of the graph convolutional network (GCN) was proposed recently and preferred to deal with graph correlations between image samples. Moreover, implementing diabetic retinopathy grading without annotations should exploit the inherent similarities from retinal images and rely heavily on correlations between their feature representations. Therefore, this study proposes a deep graph correlation network (DGCN) under an unsupervised framework, a novel deep learning technology for the automated diagnosis of diabetic retinopathy. In this study, the DGCN model was trained using unlabelled retinal images to optimize the parameters, which were randomly initialized and further explored the inherent distance correlations supervised by estimated labels. Specifically, the DGCN proposed in this study first uses a CNN as a backbone network for learning independent feature representations from retinal images conducted in a mini-batch. Then, DGCN constructs a *K* nearest neighbor graph by computing similarities to choose the top *K* samples, and forwards them into a GCN, constrained by a graph-centre (gc) loss. Through these operations, DGCN can make similar features closer to each other, and discriminant correlations in unlabelled retinal images are initially learned. Furthermore, the vital step of this study is to identify the grade for each retinal image, and DGCN designs a pairwise correlation estimating module to determine whether a pair of retinal image features belongs to the same grade, which could provide pseudo annotations automatically. Finally, the specific DGCN framework in this study integrates contrastive loss and transform-invariant constraints based on automated learned pseudo labels.

For simplicity, we summarized main symbols in this part, including retinal images *I* = {*I*_1_,*I*_2_,…,*I*_*N*_}, CNN network *r*(⋅;θ), and GCN network *G*(*F*_*b*_,*A*), where _*F_b_*_ is the CNN features in a batch, and _*A*_is the adjacent matrix for _*F_b_*_.

#### Convolutional Neural Network

The DGCN framework proposed in this study contains a joint feature learning module of a CNN and GCN, optimized by a series of loss functions, to update the network parameters.

Inspired by the excellent feature learning capability of convolutional neural network, we deploy the CNN framework to extract representative information from image pixels. Formally, retinal images are denoted by *I* = {*I*_1_,*I*_2_,…,*I*_*N*_}, and resNet-50 (29) is utilized as the backbone CNN *r*(⋅;θ). The learned feature collection of *I* can be denoted as follows:


(1) $$F=\left[F_{0},F_{1},\ldots ,F_{N}\right]=\left[r\,\left(I_{1},\theta \right),r\,\left(I_{2},\theta ,\ldots ,r\,\left(I_{N},\theta \right)\right)\right]$$

where θ denotes trainable parameters of CNN.

#### Graph Convolutional Network

To exploit the correlations between retinal images, GCN is adopted to formulate a topological structure and learn graph feature representations due to its successful application in a medical data analysis (30–32), especially for learning inherent correlations among different samples.

In a mini-batch *I*_*b*_ = [*I*_1_,⋯,*I*_*k*_,⋯,*I*_*B*_] (*B* is the batch size and 1≤*k*≤*B*), this study first builds graph correlations for feature set *F*_*b*_ = [*F*_1_,⋯,*F*_*k*_,⋯,*F*_*B*_], where *K* nearest neighbors are employed to connect to each sample. From this, DGCN connects sample *F_k_* with its *K* nearest neighbors by formulating an adjacent matrix *A* ∈ ℝ*^B×B^*,


(2) $${\text{A}}_{k\,j}=\left\{ \begin{array}{cc} 0, & \mathrm{if}\,j\in {𝒦}_{k}\\ 1, & \mathrm{if}\,j\notin {𝒦}_{k} \end{array}\right.$$

where *A*_*kj*_ denotes the correlation between *k*-th and *j*-th samples, and *k* is a collection of *K* nearest neighbors to *I_k_*.

From the learned CNN feature *F_b_* and adjacent matrix *A* in a mini-batch, *G*(*F*_*b*_,*A*) represents the graph correlations from *I_b_*. The GCN in this study consists of one input layer and *L* hidden layers (12). The graph convolution for retinal image representation is as follows:


(3) $$\begin{array}{cc} X^{\left(l\right)} & =\left[X_{1}^{\left(l\right)},\ldots ,X_{k}^{\left(l\right)},\ldots ,X_{B}^{\left(l\right)}\right]\\ & \sigma \,\left(D^{-1/2}\,A\,D^{-1/2}\,X^{\left(l-1\right)}\,W^{\left(l\right)}\right) \end{array}$$

where *l* = 0,1,…,*L*, and *D* = diag(*d*_1_,*d*_2_,…,*d*_*B*_), thus denoting diagonal matrix with $d_{k}=\sum_{j=1}^{B}A_{k\,j}\in {\mathbb{R}}^{d_{l-1}\times d_{l}}$. *X*^0^ = *F*_*b*_ as the input of the GCN, σ(⋅), which represents the activation of ReLU(⋅) = max(0,⋅), and *X*^(*l*)^ ∈ ℝ*^B×d_l_^* is the output from the *l*-th layer.

The output of the last layer *Z* = {*Z*_1_,…,*Z*_*k*_,…,*Z*_*B*_} ∈ ℝ*^B×m^* is the graph representation of the mini-batch *I_b_*, and the parameters of θ and *W* = {*W*^0^,*W*^1^,…,*W*_(*l*)_} need to be trained by several loss functions focusing on unsupervised retinal image classification.

#### Loss Function and Optimisation

One of the most critical issues of DL is the loss function, which provides directions of training and application. The loss functions of this study are as follows: gc loss, pseudo-contrastive (pc) loss, and transform-invariant loss.

This study aims to explore the correlations between retinal images, and it should make similar samples closer to network training. Thus, DGCN adopts a gc loss on graph representations in each mini-batch.


(4) $${ℒ}_{g\,c}=\frac{1}{2}\,\sum_{k=1}^{B}\sum_{j=1}^{B}A_{k\,j}\,{∥Z_{j}-Z_{k}∥}^{2}$$

where *Z_k_* is the center of *K*-nearest neighbors.

Furthermore, this study proposes a label estimator to compute pseudo-annotations for each sample. This label estimator is based on correlations between each other and only outputs pairwise annotations. Given a pair of samples *I_j_* and *I_k_* from the mini-batch, if both of them have more shared *K* nearest neighbors, it is possible that they belong to the same category. Mathematically, given *i*-th and *k*-th samples, the annotation estimation is computed as follows:


(5) $$\begin{array}{c} l\,\left(k,j\right)=\left\{ \begin{array}{cc} 0, & \mathrm{if}\,∥{𝒦}_{k}\cap {𝒦}_{j}∥\leq \lambda \\ 1, & \mathrm{if}\,∥{𝒦}_{k}\cap {𝒦}_{j}∥>\lambda \end{array}\right. \end{array}$$

where λ is a threshold to balance the number of shared neighbors.

According to Eq. 5, pairwise retinal images have contrastive annotations *l*(⋅,⋅)(0 or 1), where 1 denotes that they belong to the same category and 0 means they are in different classes. Subsequently, this study utilizes a pc loss function on each pair of samples from the mini-batch.


(6) $${ℒ}_{p\,c}=\frac{1}{2\,N}\,\sum_{\forall I_{k},I_{j}\in I_{b}}l\,\left(k,j\right)\,d^{2}+\left(1-l\,\left(k,j\right)\right)\,\text{max}\,{\left(m-d,0\right)}^{2}$$

where *d* represents the Euclidean distance and *m* is a margin parameter. This loss constraint guarantees that samples with the same pseudo pairwise label have smaller distances and those with different pseudo-annotations have larger distances.

The third concern of this study is the feature scalability of each sample after CNN and GCN; thus, a transform-invariant loss is employed to ensure that the feature representation remains invariant regardless of undergoing random transformation. Concretely, we assumed a transformation *T*(⋅) (e.g., random rotation) on each retinal image, thus generating a new image $I_{k}^{\prime }=T\,\left(I_{k}\right)$ with equal discriminative information to that of the original retinal image. It is expected that the feature representations from DGCN preserve identical information despite suffering transformations, achieved by a transform-invariant (ti) loss,


(7) ℒt⁢i=1B∑k=1B∥Zk-Z∥′k

where Zk′ represents the graph feature of Ik′.

Finally, this study employs an overall loss function to conduct optimisation for a deep GCN as follows:


(8) $$ℒ={ℒ}_{p\,c}+\alpha \,{ℒ}_{g\,c}+\beta \,{ℒ}_{t\,i}$$

where α and β are balance parameters for three terms.

### Application of Deep Graph Correlation Network Algorithm

After sufficient training for DGCN, it is then applied to retinal image grading. This study employs several gallery images $I^{g}=\left[I_{1}^{g},\ldots ,I_{i}^{g},\ldots ,I_{n}^{g}\right]$ with correct grading annotations, fed into the network and output graph features $Z^{g}=\left[Z_{1}^{g},\ldots ,Z_{i}^{g},\ldots ,Z_{n}^{g}\right]$. Given a testing retinal image *I_k_*, the DGCN obtains its annotation by


(9) $$L\,a\,b\,e\,l_{k}=\text{arg}\,\underset{c}{\text{min}}\left(Z_{k},Z_{c}^{g}\right);1\leq c\leq C$$

where $Z_{c}^{g}$ is the sample from the *c*-th class. This prediction strategy searches for the smallest distance between *I_k_* and all gallery images, and predicts its annotation using the label of the matched image.

## Results

**FIGURE 1:**
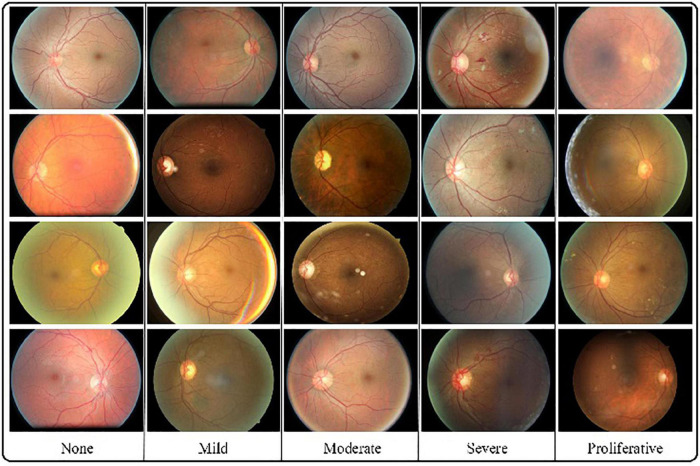
Several examples for each grade. The challenges caused by various viewpoints, illumination, and contrast can be seen in different retinal images.

In our experiments, the number of retinal images from EyePACS-1 and SXEye were 10,286 and 1,748, respectively. Each retinal image was graded by ophthalmologists 4–6 times, which were only utilized in the evaluation of this study. The EyePACS-1 data were collected from 5,158 persons, and the grading was classified into five levels: none, mild, moderate, severe, and proliferative. Specifically, EyePACS-1 consists of 2,276 (22.1%) none, 2,038 (19.8%), 1,982 (2161%) severe, and 1,829 (17.8%) proliferative. Data samples are shown in Figure 1. The deep GCN is directly trained for these two data sets when there is no need to involve manual annotations. In the evaluation, we prepared two extra data sets as a baseline with an average of 100 images from each class to calculate predictions for each retinal image, according to Eq. 9. These two baseline sets were collected from the EyePACS-1 and Messidor-2 datasets.

Table 1 reports the performance of the accuracy, sensitivity, and specificity of this algorithm. Specifically, DGCN grades EyePACS-1 with 89.9% accuracy, 88.2% sensitivity, and 91.3% specificity and Messidor-2 with 91.8, 90.2, and 93.0%, respectively. Moreover, this study compares DGCN with a retina specialist and one trained grader to conduct a comparison. From the results, DGCN performs distance of 1.8 (91.7–89.9)% accuracy, 1.3 (89.5–88.2)% sensitivity, and 1.9 (93.2–91.3)% specificity to a retinal specialist, using EyePACS as an example. It should be noted that retinal specialists require professional long-term learning and training with prohibitive cost. In contrast, DGCN is superior to short-term trained graders with approximately 1–2% advantage in each metric. From this comparison, the DGCN proposed in this study performs well with an admitted distance to a specialist, and exceeds the diagnostic capability of the trained grader. Importantly, this study does not involve manual annotations in model development, and its performance is somewhat practical for automated diabetic retinopathy diagnosis.

**TABLE 1:** Performance of sensitivity and specificity of deep graph correlation network (DGCN), compared with a retina specialist and trained graders (95% CI).

Data set	Accuracy	Sensitivity	Specificity
**EyePACS-1**	**% (95% CI)**		
Retina specialist	91.7 (88.1–93.8)	89.5 (87.2–90.6)	93.2 (88.7–94.8)
Trained grader	88.8 (84.6–92.5)	86.4 (81.2–89.8)	90.4 (87.9–92.7)
DGCN model	89.9 (87.3–91.9)	88.2 (86.4–90.0)	91.3 (89.4–93.3)
**Messidor-2**	**% (95% CI)**		
Retina specialist	93.3 (89.5–95.8)	91.0 (87.2–93.4)	95.0 (91.7–96.2)
Trained grader	89.7 (86.0–91.8)	87.9 (85.2–90.6)	91.1 (86.9–93.2)
DGCN model	91.8 (89.9–93.2)	90.2 (89.4–91.5)	93.0 (91.5–94.3)

**FIGURE 2:**
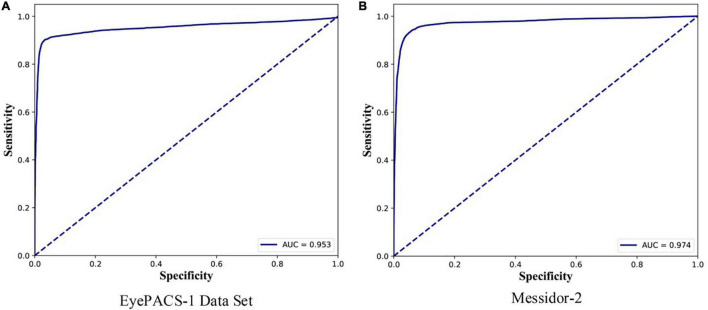
Receiver operating characteristic (ROC) curves on EyePACS-1 **(A)** and Messidor-2 **(B)** for the presence of referable diabetic retinopathy (moderate or worse diabetic retinopathy or referable diabetic macular oedema). The AUC values are shown in the right bottom area.

**FIGURE 3:**
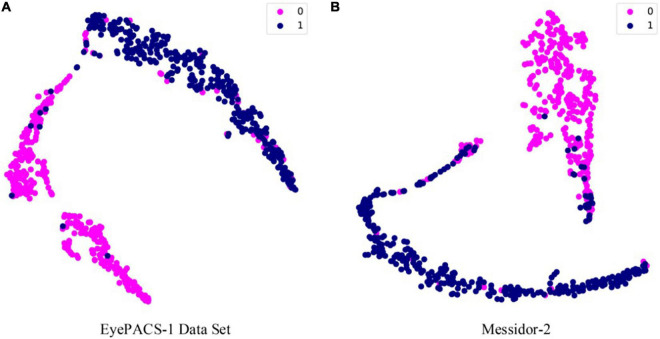
The t-distributed Stochastic Neighbor Embedding (t-SNE) performance on EyePACS-1 **(A)** and Messidor-2 **(B)**. The category division follows the ROC curve, and moderate and worse diabetic retinopathy is recognized by DR.

For the diagnosis of referable diabetic retinopathy, this study also reports the ROC curves for EyePACS-1 and Messidor-2 data sets in Figure 2, and their t-SNE plots are shown in Figure 3. DGCN achieved an AUC of 0.953 and 0.974 on EyePACS-1 and Messidor-2, respectively (Figures 2A,B). From the t-SNE performance in Figure 3, the referable diabetic retinopathy (navy points) is less misdiagnosed than normal images (deep pink points), as well as sensitivity and specificity (Table 1). Compared to the retina specialist and trained graders, the DGCN proposed in this study achieves satisfactory clinical sensitivity and specificity, as shown in Figures 2, 3 and Table 1. This demonstrates that the DGCN method can perform automated diabetic retinopathy diagnosis without manual annotations, which yields commendable results and economizes expensive well-trained professional labor.

## Discussion

The experimental results on publicly available datasets (EyePACS-1 and Messidor-2) demonstrate that automated diabetic retinopathy grading without any professional annotations is achievable, significantly alleviating economic and human resources. The performance of DGCN is close to that of a retina specialist but superior to that of trained graders. The model without training by annotations is encouraged to involve CNN and GCN to explore inherent correlations between retinal images further. It is also proven that this method is effective, with an accuracy of 89.9%, sensitivity of 88.2%, and specificity of 91.3% for EyePACS-1, and an accuracy of 91.8%, sensitivity of 90.2, and specificity of 93.0% on Messidor-2.

Inspired by successful deep learning applications in biomedical image analysis (33–35), this paper attempts to involve advanced deep learning models into retinal image classification. Considering the challenging variations in illumination, viewpoint, and contrast, retinal image grading is a severe automated diagnosis problem, even with professional annotations. Utilizing correlations between each image appearance feature is of utmost importance, and keeping the feature representation invariant when it suffers from transformations positively affects automated diabetic retinopathy grading. Aiming at these two points, this study employs a CNN to learn independent features from raw retinal images. They are then fed into a GCN with an established graph from its *K* nearest neighbors. To draw similar samples closer to each other, the DGCN model attaches a gc loss on each mini-batch to achieve this goal. Furthermore, this study designs a pairwise label estimator for given pairwise retinal features and involves pseudo-labels into a pc loss on each pair of samples. DGCN also realizes the transformation-invariant characteristics of the network by a transformation-invariant loss to preserve identical information. Finally, this study calculates the distances between each sample to a few previous samples. This contains precise annotations and determines the category by annotating the matched sample, which has the smallest distance to the target images. Compared with existing DR grading methods, the advance of our DGCN is that it has excellent capability to automatically learn discriminative information from unlabeled data, which is the easiest data to obtain.

Although the DGCN model performs well in automated diabetic retinopathy diagnosis without the aid of professional annotations, it remains a weakness for models trained by full labels. This method also has limitations on the DR grading task, including redundant training times, guidance of annotated data in applications, and lower sensitivity performance. In general, this study opens a novel economical method for automated disease diagnosis without needing professional manpower. It may produce more practical performance when utilizing few annotated data and large-scale unlabelled samples, which will be exploited in future studies.

This study elaborates on the effectiveness of deep learning technology in automated diabetic retinopathy diagnosis without manual annotations, rather than fully labeled data, as in previous methods. The feasibility of this research provides a novel ideology for computer-aided systems with economic and easy operability.

## Data Availability Statement

The original contributions presented in the study are included in the article/supplementary material, further inquiries can be directed to the corresponding authors.

## Author Contributions

GZ and BS conceived, designed and performed the analysis and wrote the manuscript. ZC contributed to the data and analysis tools and wrote the manuscript. YG performed the analysis and wrote the manuscript. ZZ collected the data and performed the analysis. KL and WY collected the data, wrote the manuscript, and supervised. All authors contributed to the article and approved the submitted version.

## Conflict of Interest

The authors declare that the research was conducted in the absence of any commercial or financial relationships that could be construed as a potential conflict of interest.

## Publisher’s Note

All claims expressed in this article are solely those of the authors and do not necessarily represent those of their affiliated organizations, or those of the publisher, the editors and the reviewers. Any product that may be evaluated in this article, or claim that may be made by its manufacturer, is not guaranteed or endorsed by the publisher.
